# Evidence of the Importance of Host Habitat Use in Predicting the Dilution Effect of Wild Boar for Deer Exposure to *Anaplasma* spp

**DOI:** 10.1371/journal.pone.0002999

**Published:** 2008-08-20

**Authors:** Agustín Estrada-Peña, Pelayo Acevedo, Francisco Ruiz-Fons, Christian Gortázar, José de la Fuente

**Affiliations:** 1 Department of Parasitology, Veterinary Faculty, Zaragoza, Spain; 2 Instituto de Investigación en Recursos Cinegéticos IREC (CSIC–UCLM–JCCM), Ciudad Real, Spain; 3 Wildlife Ecology and Management Group, Central Science Laboratory, Sand Hutton, York, United Kingdom; 4 Department of Veterinary Pathobiology, Center for Veterinary Health Sciences, Oklahoma State University, Stillwater, Oklahoma, United States of America; University of Utah, United States of America

## Abstract

Foci of tick-borne pathogens occur at fine spatial scales, and depend upon a complex arrangement of factors involving climate, host abundance and landscape composition. It has been proposed that the presence of hosts that support tick feeding but not pathogen multiplication may dilute the transmission of the pathogen. However, models need to consider the spatial component to adequately explain how hosts, ticks and pathogens are distributed into the landscape.

In this study, a novel, lattice-derived, behavior-based, spatially-explicit model was developed to test how changes in the assumed perception of different landscape elements affect the outcome of the connectivity between patches and therefore the dilution effect. The objective of this study was to explain changes in the exposure rate (ER) of red deer to *Anaplasma* spp. under different configurations of suitable habitat and landscape fragmentation in the presence of variable densities of the potentially diluting host, wild boar. The model showed that the increase in habitat fragmentation had a deep impact on Habitat Sharing Ratio (HSR), a parameter describing the amount of habitat shared by red deer and wild boar, weighted by the probability of the animals to remain together in the same patch (according to movement rules), the density of ticks and the density of animals at a given vegetation patch, and decreased the dilution effect of wild boar on deer *Anaplasma* ER.

The model was validated with data collected on deer, wild boar and tick densities, climate, landscape composition, host vegetation preferences and deer seropositivity to *Anaplasma* spp. (as a measure of ER) in 10 study sites in Spain. However, although conditions were appropriate for a dilution effect, empirical results did not show a decrease in deer ER in sites with high wild boar densities. The model showed that the HSR was the most effective parameter to explain the absence of the dilution effect. These results suggest that host habitat usage may weaken the predicted dilution effect for tick-borne pathogens and emphasize the importance of the perceptual capabilities of different hosts in different landscapes and habitat fragmentation conditions for predictions of dilution effects.

## Introduction

The genus *Anaplasma* (Rickettsiales: Anaplasmataceae) contains tick-borne pathogens that are found exclusively within membrane-bound inclusions or vacuoles in the cytoplasm of both vertebrate and tick host cells [1], [2]. *A. marginale* is host-specific for ruminants while *A. phagocytophilum* infects a wide range of hosts including rodents, ruminants, birds, felids, horses and donkeys, dogs and humans. *A. marginale* is distributed worldwide in tropical and subtropical regions of the world where it causes bovine anaplasmosis [2]. *A. phagocytophilum* is the causative agent of tick-borne fever (TBF) in ruminants and human, equine and canine granulocytic anaplasmosis [1]. Vertebrate hosts and male ticks develop persistent infections with *Anaplasma* spp. which, in turn, allows them to serve as a reservoir of infection. As they become persistently infected, vertebrate hosts remain seropositive for most of their life. *Anaplasma* are transmitted horizontally by ixodid ticks while transovarial transmission does not appear to occur. Transtadial transmission occurs from stage to stage (larvae-to-nymphs, nymphs-to-adults and larvae-to-adults). Therefore each tick generation must acquire infection by feeding on infected hosts. The tick midgut is the first site of infection in which large membrane bound vacuoles or colonies first contain reticulated forms that divide by binary fission and then subsequently transform into infective dense forms [2]. The salivary glands are then infected from where the pathogen is transmitted to new hosts [2]. The broad geographic distribution, as well as the clinical and host tropism diversity of *A. phagocytophilum* strains suggests the presence of complex infection-transmission networks that may influence the epizootiology of the disease [3].

Several genera of Ixodidae, like *Boophilus*, *Dermacentor*, *Hyalomma*, *Ixodes* and *Rhipicephalus* may be involved in the effective transmission of *Anaplasma*. Results from a previous study provided information about the evolution of *A. marginale* strains using MSP1a repeats sequences [4], corroborated its genetic heterogeneity at a global scale and suggested a tick-pathogen co-evolution. Tick larvae generally feed on rodents and other small mammals, which may be infected with *A. phagocytophilum* but not with *A. marginale* [3]. Nymphs and adults feed on large mammals such as Iberian red deer (*Cervus elaphus hispanicus*) and European wild boar (*Sus scrofa*), which may be infected with *A. phagocytophilum* and/or *A. marginale* [3]. Therefore, the most likely tick stages to transmit these pathogens are nymphs and adults for *A. phagocytophilum* and adults for *A. marginale*. Studies of ticks parasitizing on Iberian red deer and European wild boar revealed the presence of tick species that can act as vectors of *Anaplasma* spp. in south-central Spain [5]–[7]. Infections by *Anaplasma* spp. have been reported in red deer in that area [3], [5], [7]. However, although *A. phagocytophilum* DNA has been detected with low prevalence in wild boar in other European countries [8], wild boar are not infected with *Anaplasma* spp. in south-central Spain and are therefore considered to be a refractory host for these pathogens [7].

Iberian red deer and European wild boar are among the most important big game species in Spain and other European countries. Red deer is irregularly distributed in Spanish mainland, with higher densities in the south-west. Wild boar is more widespread [5], although the densities vary between high population densities reported in the northeast and in south-central Spain, and the low densities of the northern plateau and south-eastern Spain [6], [7]. Big game species are increasingly managed due to the economic importance of hunting. For this reason, fencing and artificial feeding are becoming common practices in many areas of Spain. These factors lead to elevated deer and boar densities [8], [9].

In a number of tick-pathogen systems, certain tick hosts do not support multiplication of the pathogen. Incompetent hosts may play a crucial role determining the infection prevalence in the vectors. It has been proposed for natural communities [10] that the abundance of hosts inefficient in the transmission of the pathogen to a feeding vector could act as a diluting factor in the dynamics of pathogen transmission, therefore reducing the exposure rate (ER) in competent hosts. In the case of Lyme disease, deer are refractory to infection but feed a large number of adult ticks [11]. One hypothesized form of disease control is to exclude deer from defined areas [12] which presumes that deer removal will prevent tick life cycle from being completed, thus leading to fewer ticks feeding on woodland rodents, which are the most competent hosts for the pathogen. In the case of the *Anaplasma* spp.-deer/boar system in Spain, both host species support feeding of tick nymphs and adults but only deer support the multiplication of *A. marginale* and *A. phagocytophilum*. Therefore, the presence of boar could potentially dilute the ER in deer by “removing” ticks from feeding upon deer and decreasing the force of pathogen transmission.

The transmission of tick-borne pathogens depends upon a complex arrangement of factors involving climate, tick and host abundance and landscape composition. The objective of this study was to develop a novel, lattice-derived, behavior-based, spatially-explicit model to explain changes in the ER of red deer to *Anaplasma* spp. under different configurations of suitable habitat and landscape fragmentation in the presence of variable densities of the potentially diluting host, wild boar. These studies evidence the importance of host habitat usage in predicting the dilution effect for tick-borne pathogens and could have important implications for the control of *Anaplasma* spp. infections in wild hosts to reduce potential transmission to domestic animals and humans.

## Results

### Development of a model for deer *Anaplasma* spp. ER in the presence of different densities of wild boar

A novel, lattice-derived, behavior-based, spatially-explicit model was developed to explain changes in deer *Anaplasma* spp. ER under different configurations of suitable habitat and landscape fragmentation in the presence of variable densities of the potentially diluting host, wild boar.

**Figure 1:**
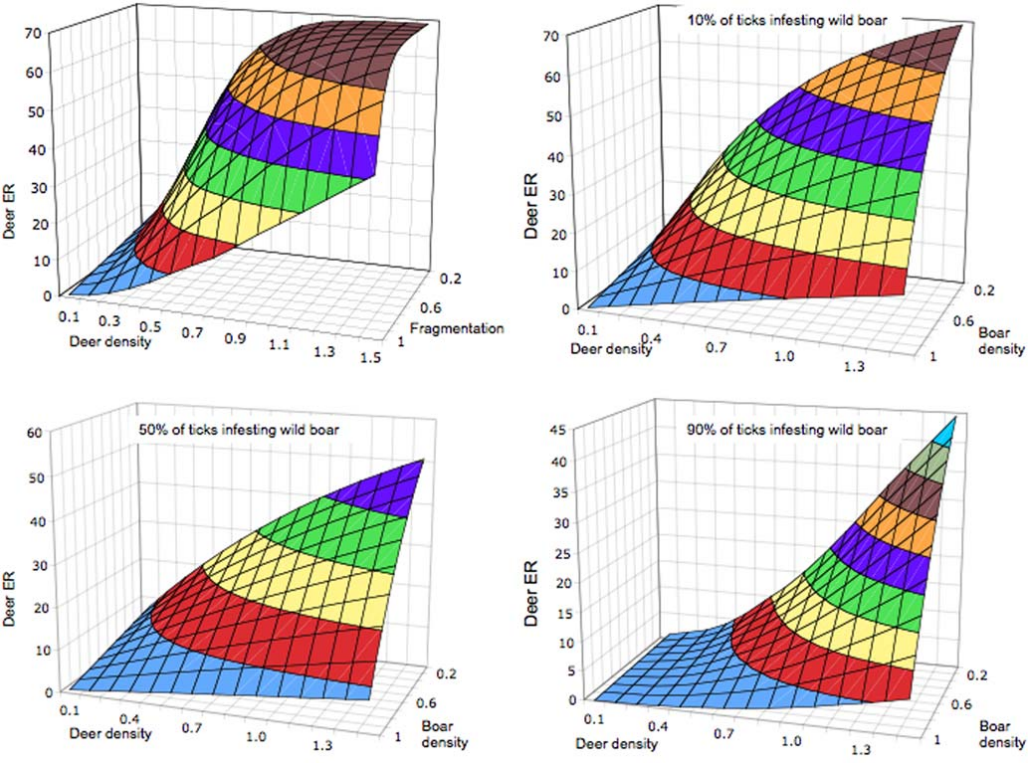
Effect of different parameters on deer *Anaplasma* spp. ER. (A) Analysis of deer ER as single host, under variable conditions of habitat fragmentation. (B–D) Effect of wild boar and deer density on deer ER at estimated 10%, 50% and 90% of ticks infesting wild boar.

The model showed that in the absence of wild boar, the increase of deer densities resulted in increased ER, while the increase of habitat fragmentation clearly decreased ER values (Fig. 1A). In the absence of wild boar, the results of the sensitivity analysis showed that deer density had the highest significant effect, while habitat perception and fragmentation had a smaller but significant effect (Table 1). Deer ER was not sensitive to some values taken as constant in the final model such as pathogen transtadial transmission and infected host-tick transmission rates.

**Table 1:** Results of sensitivity analysis performed on deer ER and HSR obtained from simulated landscapes and study sites.

Output parameter	Input parameter	Sensitivity index
ER on simulated landscapes (only deer)	Deer density	3.14
	Habitat fragmentation	1.29
	Habitat perception	1.34
	Tick transtadial transmission	0.68
	Tick transovarial transmission	0.66
	Infectivity of deer to ticks	0.21
HSR on simulated landscapes	Deer and boar density	0.98
	Habitat fragmentation	3.2
	Habitat perception	1.8
ER on simulated landscapes (deer and boar)	Deer and boar density	3.97
	HSR	3.92
	Tick preferences to hosts	1.24
ER on actual sites	Deer and boar density	not applicable
	HSR	4.1
	Tick habitat suitability	0.88
	Habitat fragmentation	1.18
	Patch size	1.19
HSR on actual sites	Habitat perception	4.12
	Fragmentation	3.76

**Figure 2:**
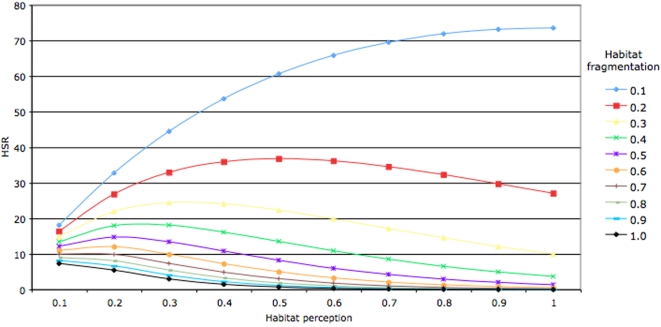
Effect of habitat fragmentation on HSR. The effect of habitat fragmentation (from 0.1, minimum assayed, to 1.0 maximum assayed) on HSR was modelled with respect to habitat perception by hosts.

The introduction of variable densities of wild boar produced a significant decrease in deer ER (Figs. 1B–D). The decrease in deer ER was evident at different wild boar tick infestations rates. However, this reduction was strongly sensitive to the Habitat Sharing Ratio (HSR), a parameter describing the amount of habitat shared by red deer and wild boar, weighted by the probability of the animals to remain together in the same patch (according to habitat perception rules), the density of ticks and the density of animals at a given vegetation patch (Table 1). The HSR was inversely proportional to the habitat perception by hosts, a measure of dwelling time of a host according to patch size and distance to another suitable patch (data not shown). The rise of habitat fragmentation had a deep impact on HSR (Fig. 2).

In summary, the model showed that deer ER decreased with increased densities of wild boar but this effect was affected by HSR (Table 1).

### Application of the model to empirical data

**Figure 3:**
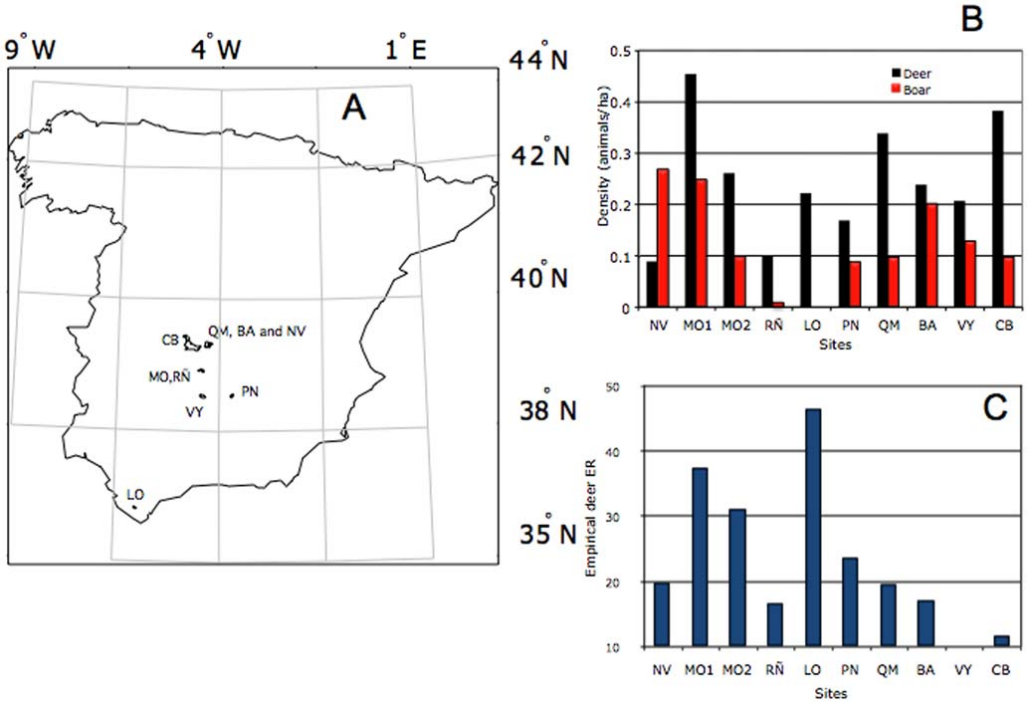
Empirical data collected from study sites. (A) Map of Spain with the location of the study sites. (B) Wild boar and deer densities on each study site. (C) Deer seropositivity to *Anaplasma* spp. was determined as a measure of ER in each study site.

Deer *Anaplasma* spp. seropositivity values were used to define ER in study sites. Deer and wild boar host densities (in animals/ha) and deer *Anaplasma* spp. ER were determined in 10 study sites in Spain (Fig. 3A). Study sites differed in wild boar and deer host densities. Deer *Anaplasma* spp. ER ranged from 10% to more than 45% in study sites (Fig. 3C). While higher values were obtained from sites where wild boar is absent (LO) or where red deer has high densities (MO), a direct relationship between red deer density, red deer/wild boar ratio and deer *Anaplasma* spp. ER was not found (t-test, p = 0.239 and p = 0.401, respectively) (Fig. 3B). Furthermore, habitat fragmentation and expected tick abundance in each site had no influence on observed ER rates (t-test, p = 0.189 and p = 0.209, respectively). Therefore, empirical data failed to show a dilution effect of wild boar on deer *Anaplasma* spp. ER.

**Figure 4:**
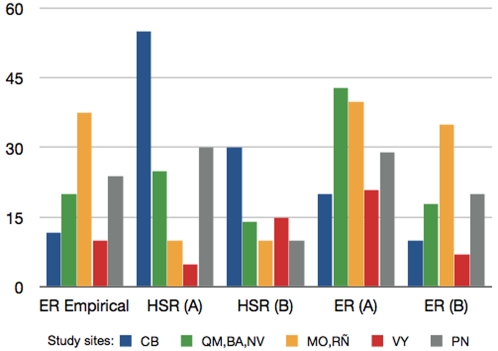
Changes in HSR lead to variable deer ER outputs. Modelled HSR and deer ER values were compared when obtained (A) under a common set of host habitat perception rules or (B) after re-distribution of hosts along vegetation patches according to empirical counts of animals at each study site. The results showed that modelled and empirical deer ER values were similar for conditions in (B) only.

The study sites had a fragmentation rate ranging from 0.5 to 1 (data not shown). To check for differences in habitat perception by each host (e.g. different type occupancy in each site) we tested for significant differences in host densities at the vegetation classes available on each site. Factorial ANOVA showed that both deer and wild boar had variable preferences towards vegetation classes in each site (p = 0.0139) and that the concentration of animals on each vegetation class was independent of the average size of the given class (p = 0.412) or its fragmentation (p = 0.308). Consequently, deer ER values obtained after re-distribution of hosts along vegetation patches according to empirical counts at each study site were similar for modelled and empirical data, thus validating the model (Fig. 4). The sensitivity analysis of these results showed that ER was most sensitive to changes in HSR (sensitivity index 4.1; Table 1), which in turn was highly dependent on habitat perception by host (including habitat type occupancy) and landscape fragmentation. Thus, small changes in HSR may lead to highly variable outputs of ER and may explain the absence of dilution effect in study sites.

## Discussion

This paper describes the first quantitative synthesis and field application of models on the dynamics of *Anaplasma* infection and the estimation of tick abundance. Basically, these models predict abundance of ticks based on climate features and landscape configuration and of pathogen dynamics within a tick-host population, respectively. Herein, we incorporated a spatial component to these models to examine the dilution effect of a potentially diluting host, wild boar, on deer *Anaplasma* ER. The model developed was validated with empirical data and showed that the dilution effect was strongly sensitive to a combination of factors related to HSR, the perception of habitat by hosts and their movement across the modelled lattices.

The results reported here showed that the increase of wild boar densities, tick preferences towards this host and HSR consistently led to a decrease in deer ER. As hosts tend to remain together in the same patch (i.e. because it has sufficient resources to support both hosts) and ticks are abundant in the same patch, the dilution effect of wild boar increases with animal density. However, habitat fragmentation leads to a clear decrease of the dilution effect. Deer ER values were shown to be slightly sensitive to parameters such as transtadial transmission and transmission rates from infected host to ticks feeding on it. Accordingly, these values were kept constant in the model following published reference values [2], [17], [18] and after preliminary runs of the lattice-derived models. Interestingly, the model was also relatively insensitive to changes in tick densities alone, demonstrating that the over dispersed distribution of ticks in the habitat patches and on their hosts (with a variance much greater than the mean) is an important factor in transmission [19].

Tick-borne diseases tend to concentrate in infection foci that occur at fine spatial scale. While the presence or even abundance of ticks can be approximated using proxy variables [20] the fine scale dynamics of tick-borne diseases is delineated by the complex interactions of landscape, climatic variables and host abundance. The spots of tick-borne pathogens are thus extremely focal in nature and tend to be obscured by an interconnected network of both abiotic and biotic factors. It has been reported that the exclusion of the main host in a natural pathogen-tick-host system has the consequence of increasing tick availability for the reservoir host, thereby increasing tick-borne pathogen prevalence [21], [22].

The results from our study suggested that although wild boar may be a potentially diluting host for deer *Anaplasma* ER, empirical results did not show a decrease in deer ER in study sites with high wild boar densities. The model showed that the HSR was the most effective parameter to explain the absence of the dilution effect, thus suggesting that host habitat usage may weaken the predicted dilution effect for tick-borne pathogens and emphasize the importance of the perceptual capabilities of different hosts in different landscapes and habitat fragmentation conditions for predictions of dilution effects.

The statistical analysis of host densities in study sites showed that hosts exhibited different preferences towards vegetation classes at each site. In general terms, habitat use is determined by food availability, shelter and weather conditions [23], [24] and these host species select habitats that offer high-energy food and cover from predators [25]. However, other characteristics such as human disturbances, population density or interspecific relationships of each population may modulate habitat use [26]. These factors are difficult to determine and may affect host habitat perception at each study site. Therefore, a common set of animal preferences for vegetation classes cannot be derived and applied to every study site. Understanding how animals disperse is a major issue for the management of tick-borne diseases.

It is obvious that landscape heterogeneity and fragmentation affect how organisms are distributed in the landscape [27], [28]. These habitat-induced changes have also deep effects on the infection rates of ticks and ER in main hosts for a tick-*Anaplasma* system, patchiness decreasing the dilution effect observed by the introduction of a refractory host. However, our system focused on large mammal hosts, which prefer relatively large habitat patches [23], [24]. Therefore, the theoretical effect of habitat fragmentation on the dilution effect as observed in the model, may be drastically different for other tick-pathogen systems, with small mammals and/or birds involved as hosts. The reduction of the dilution effect must to be considered as related to the particular system tested here, and not generalized to other tick-borne pathogens. From a practical point of view, this study demonstrates that the presence of wild boar has potential effects on the reduction of deer *Anaplasma* spp. ER. However, the high sensitivity of the model to changes in HSR under field conditions suggest that hosts habitat preferences and habitat fragmentation may affect the potential dilution effect and need to be considered for the control of *Anaplasma* spp. infections in wild hosts to reduce potential transmission to domestic animals and humans. These results may have further implications for the development of models explaining the dynamics other tick-transmitted pathogens and for the analysis of dilution effect in other systems.

## Materials and Methods

### Experimental design

A lattice-derived, spatially explicit model was developed to explain the dynamics of ticks, red deer and their *Anaplasma* spp. ER according to a wide range of landscape configurations and host densities. Then, wild boar populations were entered into that framework to predict the potential dilution effect of this species on red deer ER. Modelled results were then compared to empirical ER values (determined as deer *Anaplasma* spp. seropositivity values) collected in 10 study sites in south-central Spain (Fig. 3). These sites are fenced and contain both red deer and wild boar at variable densities, living in large areas of natural environment. Some of them are protected areas, while others are sites with hunting activities. Samples were taken during the normal hunting seasons (November–February of 2006). These large beats involve up to 100 hunters and several groups of dogs, covering areas of over 400 hectares.

### Model of tick demography

The model for tick dynamics, without any infective agent, was first developed considering deer as the only host for ticks. In this step, we fully adhered to a previously developed model [14], [15]. Briefly, the variables of this model were densities of questing and feeding tick larvae, nymphs and adults. Encounters between questing ticks and hosts are governed by mass-action. A tick-host encounter results in the transition of the tick to the feeding stage, with a certain probability of molting success after feeding. The performance of the tick population (e.g. the production of new individuals as output of the current generation) is governed by mechanisms of density-dependent regulation, according to host resistance, and operating on feeding success. A detailed, complete description of the equations governing these tick dynamics processes is given in ref. 14. These dynamics were developed through a continuous model, being the time unit one generation of ticks, thus disregarding seasonality. To simplify the model, it is assumed that the relative densities of hosts are temporally and spatially consistent. Transmission rates are thus accumulated for one complete tick generation, with independence of the moment of the year parasitic stages are more abundant. The model is accordingly aimed to explain the transmission rates, not the seasonality of the ticks in a given area. The final set of model parameters is included in table 2.

**Table 2:** Parameters of the model and ranges tested.

Model	Number	Parameter	Values	Comments or references
Simulated landscapes	1	Percent of suitable habitat for deer or boar	50%	Kept constant after preliminary runs
	2	Habitat fragmentation	5	Varied from 0 to 1
	3	Deer and boar densities	1 per ha	Varied from 1 to 15
	4	Initial number of female ticks	10 per ha	
	5	Optimum attractiveness of patch size for deer	1 ha	Varied from 0.5 to 100 ha
	6	Optimum attractiveness of patch distance for deer	100 m (500 m for boar)	Varied from 50 to 1000 m
	7	Percent of ticks infected while feeding on infected deer	50%	Ref 7
	8	Transestadial transmission	90%	Ref 7
	9	Transovarial transmission	0%	Ref 7
	10	Percent of infected deer at the beginning of simulation	10%	Kept constant
	11	Preferences of ticks towards deer and boar		Varied from 10% to 90%
Study sites	1, 2		Obtained from remote sensing features on Landsat imagery	
	3		Obtained from field counts	
	4		Obtained from a model of tick habitat suitability and abundance	Refs 14, 37
	5 to 10	As in simulated		
	11	Kept constant at 50/50		

The model of tick dynamics was spatially explicit, being developed in a lattice of patches of variable size, considered as habitat or non-habitat for the red deer. Equations governing tick abundance were calculated for every patch of the lattice, according to the host abundance in every single patch. Lattices with a random distribution of patches of different sizes were generated according to a normal distribution. SimMap [29] was used to produce “landscapes” of variable patch number and size, randomly located according to a normal distribution. It also provided with an estimation of the habitat fragmentation [30], varying between 0 (there is only 1 patch in the landscape) to 1 (maximum patchiness). Hosts were assumed to move across the landscape according to the shape and size of every patch and a set of rules involving habitat perception [31], [32]. When host densities were higher in a given patch, the probability of tick-host encounters increased and the tick population had a lower mortality in the questing phase and an increase in the rate in which ticks progressed from stage to stage and reproduced. However, as tick population performance decreases with tick density, equilibrium density of the tick population will saturate with increasing host density.

After producing a lattice with random patches and 50% of patches suitable for deer colonization, hosts were allowed to disperse across the environment. Dispersal started from an initial, randomly selected habitat patch. The transition probability from patch i to patch j via frontier ij depended on the habitat perception rules. According to graph theory [32], the probability that an individual in node i will disperse to node j can be expressed in the form of a flux rate or dispersal probability matrix. Thus, the expected dispersal flux from patch i to j is:
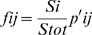
where Si is the area of patch i, Stot is the sum of the areas of every available patch, and p′ij is the probability of dispersal from i to j. This probability of dispersal p′ij is directly related to the area of patches i,j, and inversely related to the distance between them. Total traversability is thus defined as the sum of partial dispersal flux probabilities for every link, as a measure of the permeability of that patch to propagules coming from different patches in the network of the landscape [32]. To define p′ij we used a function, called habitat perception, of the form:
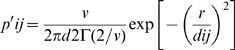
where dij is the distance between the centroids of patches, r the mean dispersal distance of the host (km) and Γ the gamma function. The parameter v relates the proportion of dispersing hosts as a response to the patch size. It has been assumed that proportion of moving hosts has a simple inverse relationship with patch size (i.e. small patches allow high migration rates) and the v parameter is simply a modifier of such a response in the gamma function.

**Figure 5:**
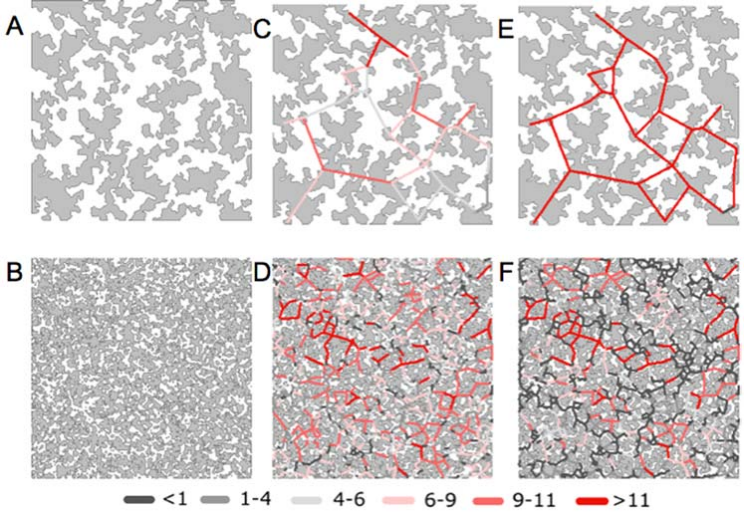
The process of creation of two lattices with high (A) and low (B) habitat fragmentation for the development of the base parameters in the tick-host model. The amount of suitable habitat (patches) is the same for both lattices (50%), and only fragmentation is changed (A: 0.8; B: 0.3). The resulting dispersal flux (patch traversability) of hosts in these lattices is included as colour lines depicting traversability from patch to patch, assuming maximum attractiveness for medium sized patches (10 ha) located at a distance of 100 m (i.e. low habitat perception, C, D) or maximum attractiveness for a small patch (0.1 ha) located at a distance of 1000 m (i.e. high habitat perception, E, F).

**Figure 6:**
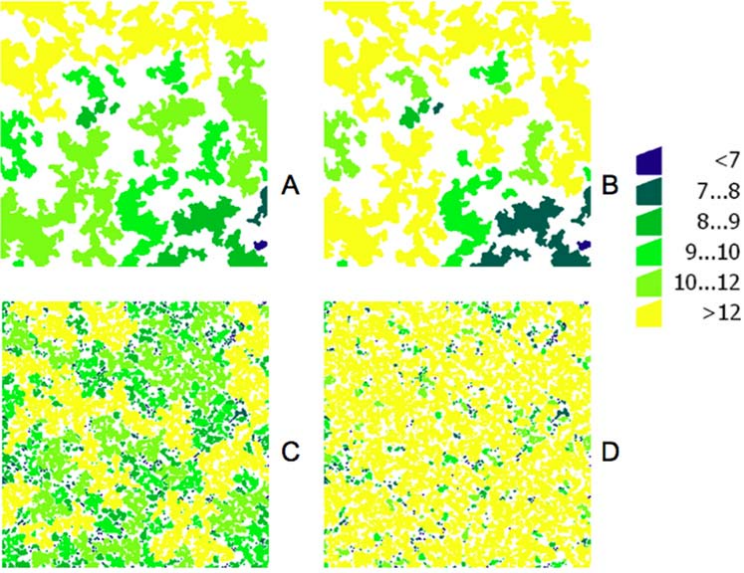
Animal densities as derived from the lattices in Figure 5, and expressed as number of hosts/ha for (A) low fragmentation/low habitat perception, (B) low fragmentation/high habitat perception, (C) high fragmentation/low habitat perception, and (D) high fragmentation/high habitat perception. High traversability conditions resulted in the accumulation of hosts in some patches, even under conditions of high habitat fragmentation.

Figures 5 and 6 illustrate the steps taken for the development of lattices and the influence of the different parameters involved on habitat perception by hosts, traversability and animal densities. After preliminary runs, it was assumed as a base parameter that maximum attractiveness for deer was a patch of 1 ha located at 100 m. Basic equations governing tick abundance were then applied to every patch in the lattice according to host densities.

The model was run with different values for tick density-dependent regulation to obtain a stable population of ticks after 50 generations. The parameters of the final, stable model are summarized in Table 1. The model was initially run with a habitat fragmentation of 0.5 and a deer density of 0.1 animals/ha, with an initial tick density of 10 engorged females/ha. These parameters produced permanent tick populations after 50 runs under realistic conditions of animal densities and habitat fragmentation.

### Model of *Anaplasma* spp. infections in red deer

The set of equations governing tick dynamics was used to model *Anaplasma* spp. ER in red deer. It has been previously demonstrated that only systemic transmission of *A. marginale* does occur [18]. Ticks feeding on infected deer may become infected with the pathogen. The model equations [15] divided deer and all tick stages into susceptible and infected. After molting, ticks remain infected because of transtadial transmission of the pathogen. Transovarial transmission was considered to be negligible for *A. marginale* [2]. Preliminary runs of the model were done looking for a combination of parameters concerning transtadial transmission and infectivity of hosts for ticks, allowing a stable infection rate in tick generations under different landscape configurations. These parameters showed that a stable system is obtained with an initial tick infection rates as low as 20%. The stable system obtained with these conditions incorporated a rate of transtadial transmission of 90% and an infectivity of 50% for ticks feeding on an infected host.

After the model of uninfected tick populations was run until equilibrium, an initial infection rate of 10% was introduced into the red deer population, leaving the model running for 50 additional tick generations. Changes in ER according to deer density and habitat fragmentation where obtained running sets of 50 landscapes, each set having a host density ranging from 0.1 to 1.5 animals/ha and a fragmentation ranging from 0.1 (very low) to 1 (very high).

### Model of the impact of the introduction of a potentially diluting host

The basic lattice-derived model was modified considering a two-hosts system in the equations governing tick dynamics [14], [15]. The main aim was to assess the effect of a host refractory to *Anaplasma* spp. infection, wild boar, on the deer ER. The model began by introducing into each landscape variable ratios between the density of red deer and wild boar, from 0.1 to 1.5 animals/ha. An additional parameter that regulates tick preferences towards the hosts was also introduced. Variable preferences of ticks towards each host were assumed varying from 10–90 (i.e. 10% of tick would prefer boar and 90% will prefer deer for feeding) to 90–10 (i.e. the opposite situation). Sets of 50 landscapes for each of these assumptions were run, including a 10% infected red deer after the stabilization of the tick population. A sensitivity analysis was performed [33], to evaluate the influence of input variables into the final ER. In this analysis, input parameters were the ratio between host densities and the preferences of ticks towards each host. The output parameter was the deer ER.

The relative importance of habitat fragmentation and host perception on deer ER, a new feature called the Habitat Sharing Ratio (HSR) was introduced. It was explicitly defined as the amount of habitat shared by both deer and boar, weighted by the probability of the animals to remain together in the same patch (according to habitat perception rules), the density of ticks and the density of both hosts at a given patch. The HSR for the whole landscape is the average of the partial values obtained for every patch and is dependent upon the habitat fragmentation and the perception of the habitat by each host species. A set of 50 landscapes was produced, with different fragmentation characteristics (from 0.1 to 1) and varying the host patch perception distance between 50 and 1000 m and the optimum size of the patch from 0.5 to 10 ha in the equation describing probabilities of host movement. A sensitivity analysis was conducted to check for effects of these variables on deer ER.

### Collection of empirical data on *Anaplasma* spp. ER, and deer and boar densities

Ten study sites were selected in south-central Spain (Fig. 3). A random age- and sex-stratified subset of animals was selected for analysis. Blood was collected and antibodies for *Anaplasma* spp. were determined in red deer using the competitive ELISA (cELISA) from VMRD, Inc. (Pullman, WA, USA) following the manufacturer's instructions [34]. Percent inhibition values greater than 30% were considered positive using the anaplasmosis cELISA [17].

Estimations of animal densities were obtained through censuses carried out in August-September 2006 (our of the hunting season). Spotlight counts were done to evaluate deer density, counting animals with a light onboard of a car and across a transect length of 15 km at an average speed of 10 km/h. Obtained data were processed using the software package Distance Sampling 5.0 [35] which provided an estimation of the density of animals in each site [36]. Wild boar abundance estimates were based on dropping frequency counts [7] in 40 transects of 100 m. The obtained abundance index was transformed into wild boar density by means of the following equation: boar density (animal/ha) = 0.0325+0.2515×dropping frequency (P. Acevedo, unpublished data).

### Application of the model to empirical deer *Anaplasma* spp. ER

The densities of the two tick species likely involved in the transmission of *Anaplasma* spp. in the study area, *Dermacentor marginatus* and *Hyalomma marginatum* was explained using a spatially explicit, climate-driven model that has been previously developed and tested [16], [37]. Prediction of tick abundance requires taking into account: (i) the abiotic properties of the site, which reflect how ticks can survive and populate a site and (ii) the movements of hosts through the landscape patches, which explain the densities of host populations. The persistence of ticks in any habitat will be influenced by the abiotic (climate and vegetation) suitability of the habitat and the rescue effect produced by neighbouring populations through host interchange between patches. Abiotic variables included in the model were mean, absolute maximum and absolute minimum yearly temperature, monthly mean maximum and minimum temperatures (in °C), total and mean monthly rainfall (in mm), mean, absolute maximum and minimum Normalized Derived Vegetation Index (NDVI from −1 to 1), mean monthly maximum NDVI, and a yearly plant productivity index. These variables were subjected to a maximum enthropy algorithm that produces an estimation of the habitat suitability for ticks (K) based on the calculation of the distance between the conditions preferred by the tick species and the actual set of long-term abiotic features.

The density of ticks at patch level is the term called Recruitment (R) and was defined as:
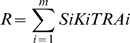
where S_i_ is the size of the patch i, K_i_ the abiotic suitability of the i^th^ patch for ticks and TRA_i_ the dispersal flux (or individual traversability) of that patch. Published reports showed that this model was able to explain much of the variation in the density of ticks in natural patches (R^2^ = 0.87, p = 0.001; see ref. 16). The model was developed with a commercially available data set of long-term monthly temperature and rainfall values for the study area at a resolution of 200 m/pixel [38], together with the NDVI and a plant-productivity index, both freely available from the NOAA-AVHRR satellites series. The computed tick density at each patch was used as input density per ha of tick females at the beginning of the simulations.

In a previous report [16], it was assumed that hosts are homogeneously distributed across the landscape. However, we incorporated into the lattice-derived model the host habitat perception towards vegetation categories and the resulting landscape network of patches to further refine the model. Vegetation categories features and fragmentation features at each site were produced through a classification of high-resolution Landsat satellite images (30 m). A total of 10 vegetation classes were extracted. For simplicity, we grouped the sites QM, NV and BA into a unique site, because they are small and contiguous. In the same way, sites MO and RÑ were also grouped. Unluckily, high-resolution satellite images available for LO were contaminated by clouds. Therefore, this site was removed from these analyses. It was assumed as a general rule for habitat perception by hosts that patches of forest and grass were suitable habitats for red deer while forest, grass and bush-shrub were suitable habitats for wild boar.

In the first simulation, the model was run using consensus parameters for all study sites and computed the traversability, tick recruitment, HSR and ER considering the general rule of habitat type preferences outlined above, distributing animal densities between these habitat types categories and according to the main rules of preferred patch size and distance. In the second simulation, we used empirical habitat type preferences towards vegetation categories, which were variable between sites, derived from field host counts, and the same parameters as before were recomputed for each site. These simulations produced different results concerning deer ER and the potential dilution effect of wild boar. A factorial ANOVA was performed using sites and vegetation classes as categorical (nested) variables being the animal density at each site the dependent variable. Differences between modelled and empirical deer *Anaplasma* spp. ER values were examined and a sensitivity analysis performed with every parameter to understand the causes of variation.
